# The Effects of Multiple Use and Sterilization on the Accuracy of Readings of Different Types of Torque Controllers

**DOI:** 10.3390/bioengineering13070715

**Published:** 2026-06-23

**Authors:** Piotr Stendera, Jolanta Kostrzewa-Janicka

**Affiliations:** Department of Prosthetic Dentistry, Medical University of Warsaw, Ul. Binieckiego 6, 02-097 Warsaw, Poland; jolanta.kostrzewa-janicka@wum.edu.pl

**Keywords:** torque wrenches, torque control, torque verification

## Abstract

Application of the appropriate torque in implant prosthodontics is very important from a clinical perspective. In implant prosthodontics, three basic types of torque wrenches are used. There are few reports in the dental literature on the possibility of controlling the torque generated by the wrench. The aim of this study was to determine the precision of measurements of various types of torque wrenches after the specified number of application and sterilization cycles. The materials consisted of 22 torque wrenches (10 spring-type, 6 toggle-type, and 6 contra-angle-type). The research methodology included measuring the accuracy of torque value indications using the gravity method. The research was conducted for different torque ranges for brand new wrenches and after 50 and 100 sterilization cycles. After each sterilization cycle, the wrenches were used to generate torque several times. Results: Before sterilization, the spring-type torque controllers were characterized by the lowest accuracy (in the largest range at low torque). The average deviation for contra-angle-type and toggle-type wrenches was smaller. After sterilization cycles, the toggle-type wrenches showed the smallest changes in measurement precision. Conclusions: The effects of multiple use and sterilization on the accuracy of readings of different types of torque controllers seem limited overall; the greatest stability of measurement accuracy was demonstrated by the toggle-type wrenches, while the spring-type wrenches presented the greatest decrease in measurement precision.

## 1. Introduction

Modern dental implantology is based on the screw fixation of both screw-retained and cement-retained restorations, where the abutment is fixed with a screw. From a clinical perspective, connection stability is crucial [1,2,3,4]. Torque wrenches (MTLDs—manual torque-limiting devices) are used for the proper control of torque when tightening the restoration’s screw. The appropriate torque is based on the manufacturer’s recommendations and is related to various clinical situations (depending on the type of restoration, screw type, material, screw surface, and even screw diameter). The application of insufficient torque may result in uncontrolled, spontaneous loosening of the screw, while tightening it with excessive torque can result in mechanical damage (fracture).

Torque is a vector quantity that is the product of force and the arm’s length, which is the distance from the point of force application to the axis of rotation. The SI unit of torque is Nm, where 1 Nm is the torque when a force of 1 N is applied perpendicularly to the arm at a distance of 1 m from the axis of rotation. Changes in torque can be achieved by changing the force or the arm’s length.

Three basic types of wrenches are used in modern implant prosthodontics: spring-type (spring-style, beam-type), toggle-type (toggle-style, friction-type, friction-style, peak-brake), and contra-angle-type. Spring-type torque controllers are equipped with a rigid arm with a scale and a spring arm [5,6,7,8,9,10,11,12]. The application of manual force deflects the spring arm; this deflection is compared to the scale and provides information on the currently applied torque. Friction-type wrenches have a scale on which the maximum torque is set [13,14]. Tracking the current torque is not possible. When the manual application of force increases, the wrench “breaks” upon reaching the set value, making further torque application impossible. Contra-angle wrenches operate similarly to friction-type wrenches. They have a scale on which the maximum torque is set, but the screwdriver is mounted (like a drill) in the head, which is similar to a slow-speed clinical contra-angle handpiece. Rotation of the wrench is achieved by turning a knob on the tip. When the set torque is reached, a clicking sound is heard, and a further increase in torque is not possible.

Implant manufacturers do not provide service for mechanical torque wrenches, and there are few descriptions in the professional literature of devices that would allow clinicians to check the accuracy of the readings [3,9,15,16,17]. According to popular opinion [2,11,18,19,20,21,22], torque controllers may become decalibrated as a result of many years of use and repeated sterilizations. As mentioned, the loss of precision of torque wrenches may be harmful from a clinical point of view.

It is also worth mentioning that during implant installation, in addition to electronic devices, manual devices are also used. Their function and design are similar to the spring-type controllers used in implant prosthodontics. These devices are also constantly used and sterilized, and their precision is crucial as well.

## 2. The Aim

The aim of this study was to determine the precision of the measurements of various types of torque wrenches after a specified number of application and sterilization cycles.

## 3. The Materials and Methods

**Figure 1:**
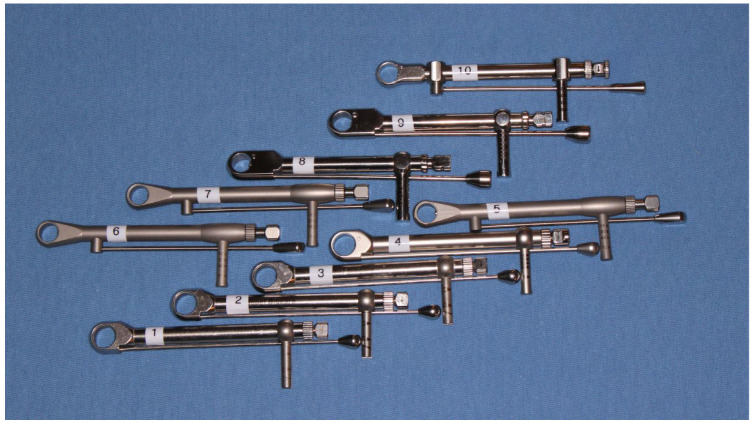
The spring-type group of torque controllers.

**Figure 2:**
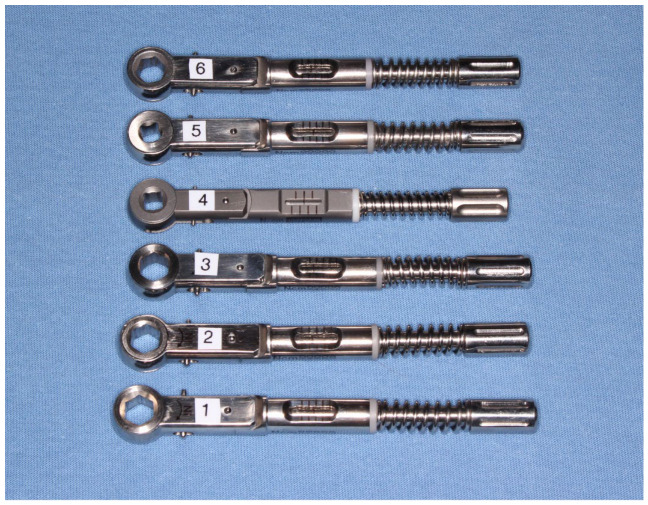
The toggle-type group of torque controllers.

**Figure 3:**
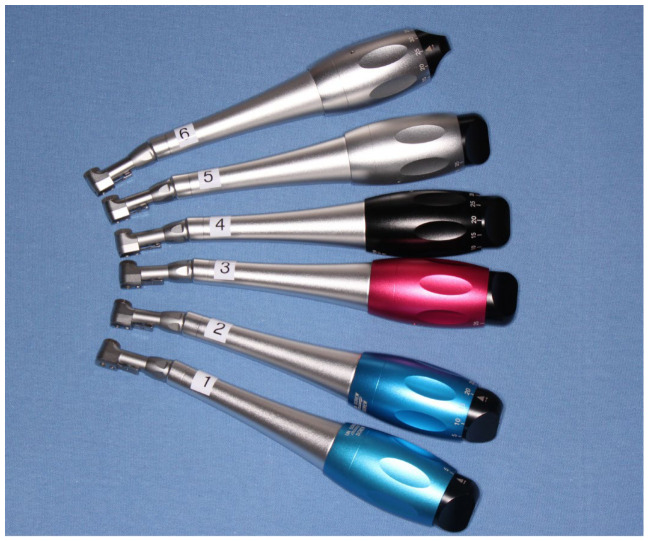
The contra-angle-type group of torque controllers.

The materials consisted of 22 new torque wrenches: 10 spring-type (3 Nobel Biocare, 3 Azdent, 2 MIS, 2 Dentmark—Figure 1), 6 toggle-type (3 Keystone, 2 Alpha-Bio, 1 Meisinger—Figure 2), and 6 contra-angle-type (Azdent—Figure 3).

The research methodology was based on measuring the accuracy of the torque value indications using the gravitational method with a modification, taking into account the specificity of the measurements for each group of keys.

All the constructed devices were based on the concept of torque calculation using the gravitational method. For each type of wrench, it was necessary to consider the specific design and function of the device.

**Figure 4:**
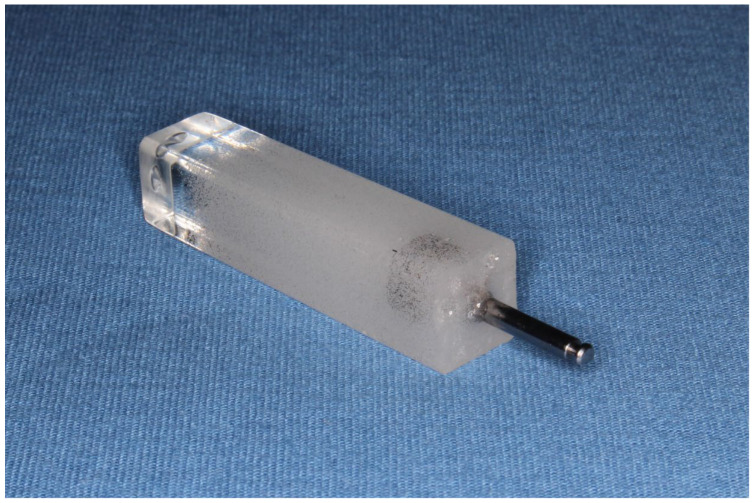
Rectangular block of epoxy resin and the handle for the rubber-gum.

**Figure 5:**
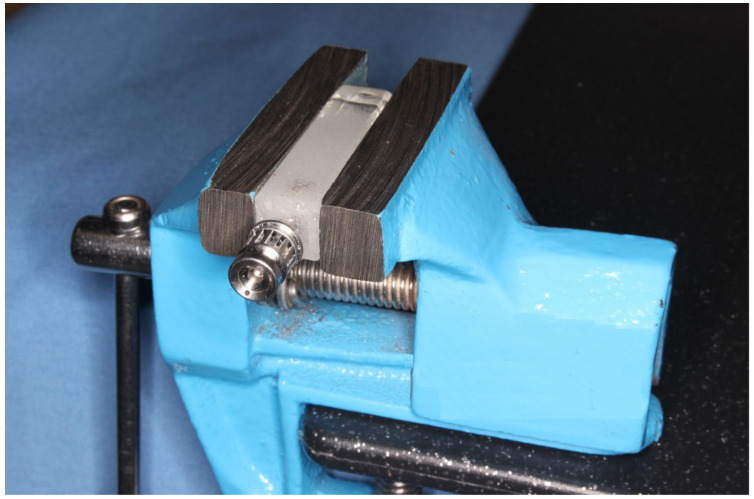
The block tightened in the clamp together with the wrench and insert.

**Figure 6:**
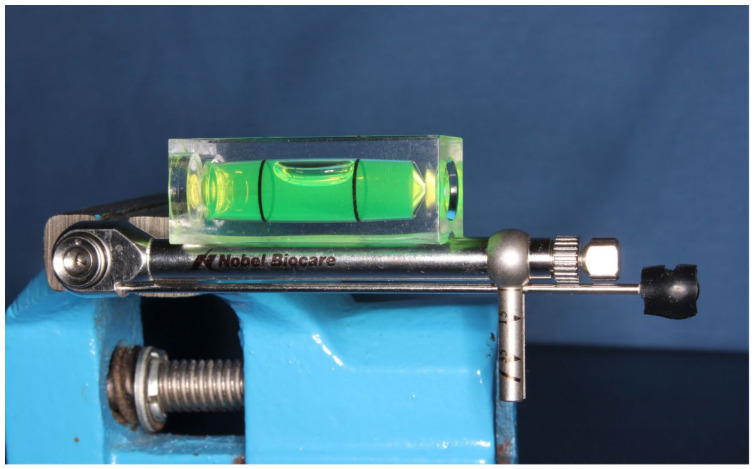
Horizontal position of the wrench arm monitored with the waterpass level.

**Figure 7:**
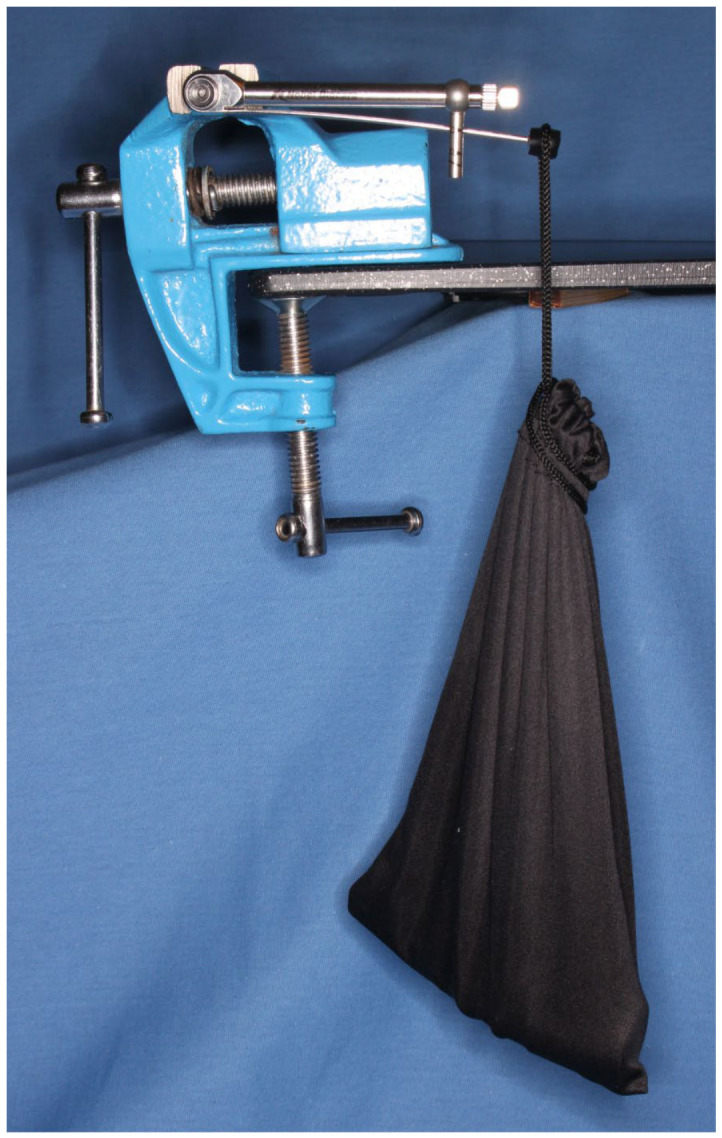
The spring arm after loading.

**Figure 8:**
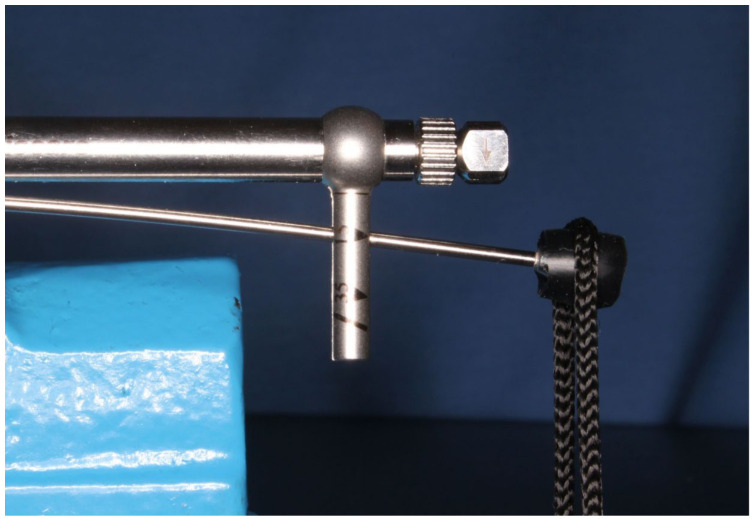
Readings on the scale of the torque controller.

For the spring-type wrench group, the research methodology involved constructing a rectangular block of epoxy resin in which the handle for the rubber-gum was placed (Figure 4). A part of the handle protruded from the block, allowing the wrench and insert to be mounted, similarly to a screwdriver (all the machine screwdrivers had the same design, like drills mounted in a clinical contra-angle handpiece). The block was tightened in the clamp (Figure 5), and the horizontal position of the wrench was monitored using the waterpass level (Figure 6). The length of the spring arm was measured with a caliper. The end of the spring arm was loaded with a bag of weights (Figure 7). The mass of the weights was gradually increased until the appropriate torque readings were reached on the wrench scale (Figure 8). The entire bag of weights was then weighed on a laboratory scale (±1 g).

**Figure 9:**
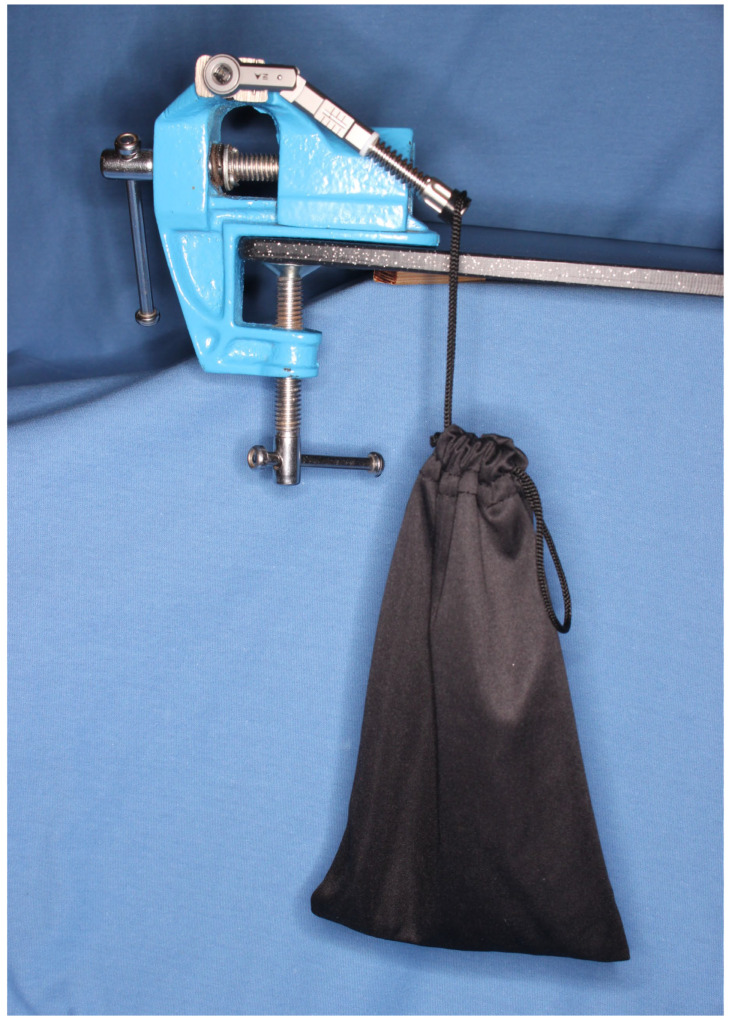
The loading of the friction-type wrench.

For the toggle-type wrenches, the test methodology was similar. The wrench was placed in a block mounted in a clamp. The length of the wrench arm was measured, its horizontal position was checked, the load was increased until the wrench clicked (Figure 9), and then the bag of weights was weighed on a laboratory scale (±1 g).

**Figure 10:**
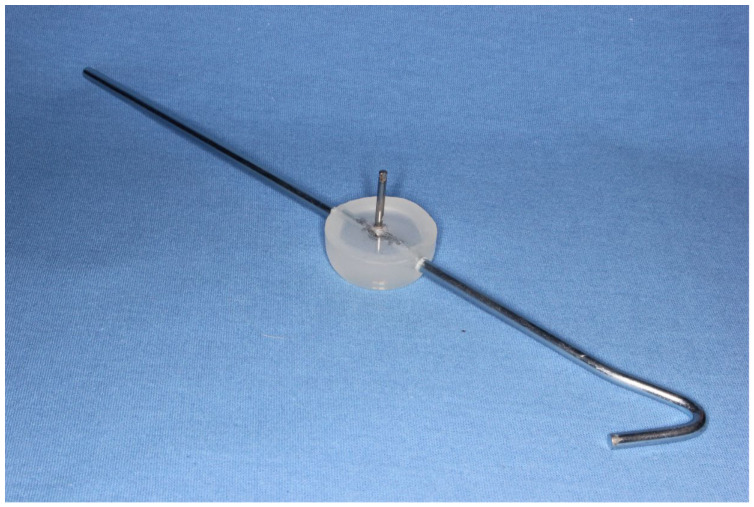
Double-sided lever made with a handle for the rubber-gum, a metal rod with a hook, and epoxy resin.

**Figure 11:**
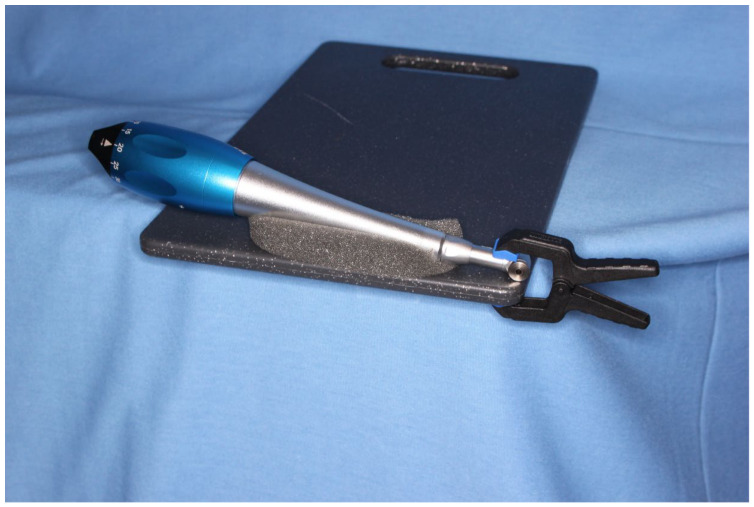
The contra-angle-type torque controller attached to the plate with the clip.

**Figure 12:**
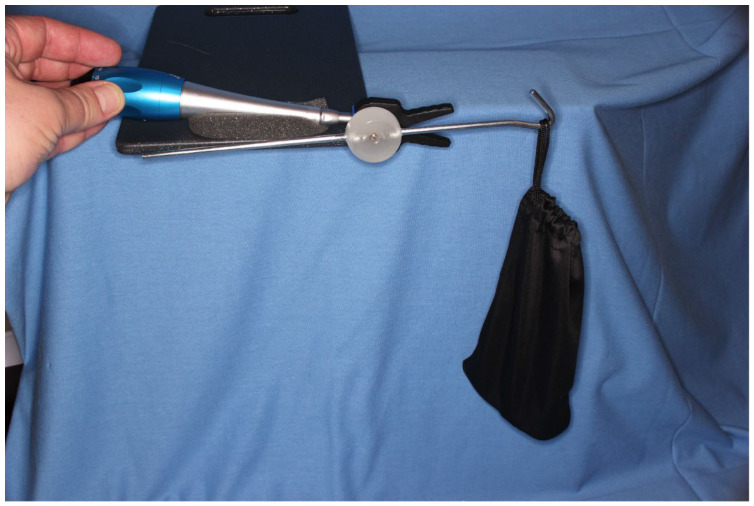
The manual torque application.

For the contra-angle-type wrenches, the test methodology had to be completely different. Using a handle for the rubber-gum, a metal rod with a hook, and epoxy resin, a double-sided lever was constructed (Figure 10). This element was balanced to eliminate the influence of the arm’s weight. The arm’s length was measured. The contra-angle-type torque controller was attached to the plate with the clip (Figure 11), and the weight was placed on the hook. The mass of the weights was increased, and a manual elevation test was performed (Figure 12). Using a laboratory scale (±1 g), the mass that caused the wrench to click when the arm was raised to a horizontal position was determined.

In order to reduce operator-dependent effects, all procedures related to the torque controllers (simulation, measurements, and weighting) were performed by one person throughout this study.

The current torque was calculated using the following formula (derived from the definition of the moment of force):

$$T\;[\mathrm{Ncm}]\;=\;r\;[\mathrm{cm}]\;\times \;m\;[\mathrm{kg}]\;\times \;9.81\;m/s^{2}\;\times \;\cos\alpha$$

where

T is the torque in newton centimeters;

r is the arm length (radius) in centimeters—the distance between the axis of rotation and the load point;

m is the mass of the weights in kilograms (including the bag or pan);

9.81 m/s^2^ is the standard gravity (gravitational acceleration), which allows for the conversion of mass to weight;

α is the angle between the wrench arm and the horizontal plane.

The wrench arm was leveled to eliminate the influence of the last factor in this equation. The cosine function value for 0 degrees is 1.

**Figure 13:**
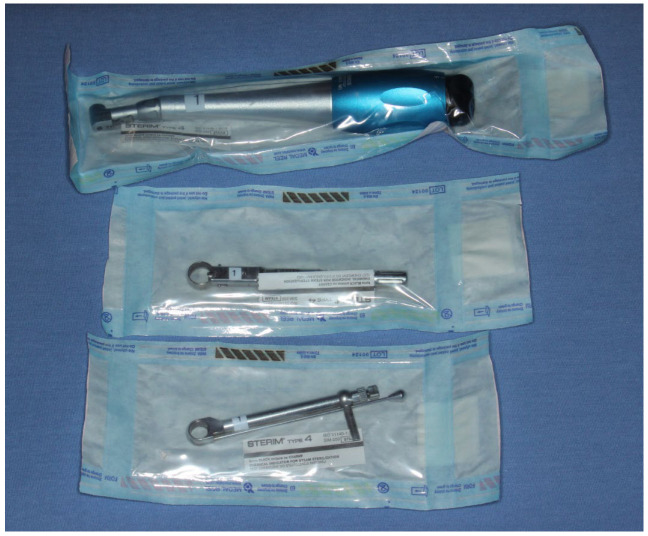
The wrenches in packages after autoclaving.

Tests were conducted for various torque ranges for brand-new wrenches before use and after 50 and 100 sterilization cycles (Figure 13). After each sterilization cycle (135 °C, 2.1 bar, 5 min), the wrenches were simulated to generate torque five times and once every 10 cycles, the wrenches were disassembled and serviced according to the manufacturer’s recommendations (cleaning, rinsing, and maintenance with spray oil for handpieces). The simulated use of torque controllers consisted of a procedure as close as possible to how the controller would be used in clinical conditions for a single patient. The screw-retained single-unit restoration on the model was unscrewed and tightened five times using the maximum torque.

After 50 and 100 sterilization cycles, testing was performed using the described gravity method. For spring wrenches, testing was performed at low (10/15 Ncm), medium (30/35 Ncm), and high (45/50 Ncm) torque values. The high value was tested only for the 7 wrenches with the wide torque range. For toggle-type wrenches, testing was performed at low (20 Ncm) and high (40 Ncm) torque values, and for contra-angle-type wrenches, testing was performed at 15 Ncm and 35 Ncm, respectively.

Descriptive statistics (mean, standard deviation, range) were calculated for torque values and percentage deviations from theoretical settings. The distribution normality was assessed using the Shapiro–Wilk test, and skewness and kurtosis were measured. Paired *t*-tests were used to compare the baseline torque with the theoretical setting and compare the torque values at baseline with the torque values after 50 and 100 sterilizations. Differences in baseline percentage deviation between wrench types were analyzed using one-way ANOVA, followed by Tukey’s post hoc test. All tests were two-tailed with a significance threshold of *p* < 0.05. Analyses were performed using R software, version 4.4.2.

The statistical analysis was based on n = 22 wrenches across three types: spring-type (n = 10), toggle-type (n = 6), and contra-angle-type (n = 6). Actual torque measurements were taken before sterilizations (baseline measurement), after 50 sterilizations, and after 100 sterilizations. In the case of each wrench, two or three settings of torque were tested.

## 4. Results

### 4.1. Baseline Agreement with Theoretical Wrench Setting

A baseline measurement was taken to verify the agreement of the actual torque with the theoretical torque setting. Descriptive statistics of the actual baseline torque by wrench subtype and type are presented in Table 1. The actual baseline measurements were also compared to the theoretical torque setting for each key, and the % difference was calculated to assess the % distance of the actual torque from the setting.

In the group of spring-type wrenches set to 10/15 Ncm, the actual torque was 16.20 ± 3.05 Ncm and ranged from 11.00 Ncm to 21.94 Ncm. The difference between the theoretical and actual torque (in %) amounted to 16.85 ± 20.94. A paired test comparing the actual torque to the theoretical setting confirmed a statistically significant difference (*p* = 0.029). In the group of spring-type wrenches set to 30/35 Ncm, the actual torque was 34.86 ± 5.02 Ncm and ranged from 30.12 Ncm to 44.58 Ncm, and in the group of spring-type wrenches set to 45/50 Ncm, it was 50.03 ± 7.14 Ncm, with a range from 41.75 Ncm to 59.64 Ncm. The difference in % indicated was weaker for higher settings of spring-type wrenches compared to the 10/15 Ncm setting (9.44 ± 16.91 and 8.02 ± 10.87 for 30/35 Ncm and 45/50 Ncm, respectively). Paired tests comparing the actual torque to the theoretical setting did not indicate statistically significant differences (*p* = 0.128 and *p* = 0.096 for 30/35 Ncm and 45/50 Ncm, respectively). Overall, paired comparisons between the theoretical and measured baseline torques for all the spring-type wrenches indicated significant differences, *p* = 0.002, with the % difference amounting to 11.82 ± 17.14.

In the group of toggle-type wrenches set to 20 Ncm, the actual torque was 20.20 ± 3.87 Ncm and ranged from 12.72 Ncm to 22.95 Ncm. The difference between the theoretical and actual torque (in %) amounted to 0.99 ± 19.36. In the group of toggle-type wrenches set to 40 Ncm, the actual torque was 37.66 ± 4.93 Ncm and ranged from 29.03 Ncm to 44.24 Ncm. The difference in % indicated weaker deviation from the setting for toggle-type wrenches set to 20 Ncm (0.99 ± 19.36 vs. −5.85 ± 12.32 for the 40 Ncm setup). Overall, the % difference for toggle-type wrenches amounted to −2.43 ± 15.88. No statistically significant difference was confirmed by paired tests, neither at the subtype nor type level (*p* > 0.05).

In the group of contra-angle-type wrenches set to 15 Ncm, the actual torque was 14.77 ± 2.22 Ncm and ranged from 12.10 Ncm to 17.40 Ncm. The difference between the theoretical and actual torque (in %) amounted to −1.56 ± 14.79. In the group of contra-angle-type wrenches set to 35 Ncm, the actual torque was 31.22 ± 5.80 Ncm and ranged from 24.90 Ncm to 41.20 Ncm. The difference in % indicated weaker deviation from the setting for contra-angle-type wrenches set to 15 Ncm (−1.56 ± 14.79 vs. −10.81 ± 16.57 for the 35 Ncm setup). Overall, the % difference in the actual value vs. theoretical setting for contra-angle-type wrenches amounted to −6.18 ± 15.73. No statistically significant difference was confirmed by paired tests, neither at the subtype nor type level (*p* > 0.05).

ANOVA confirmed significant differences between the % deviation from the theoretical setting between mechanical wrench types, *p* = 0.004. A post hoc Tukey test indicated a higher % deviation in the case of spring-type wrenches compared to contra-angle-type wrenches (*p* adj = 0.008) and in the case of spring-type wrenches compared to toggle-type wrenches (*p* adj = 0.043), while the % deviations in the case of toggle-type wrenches and contra-angle-type wrenches were statistically equal (*p* adj = 0.844).

### 4.2. Torque Evolution After Sterilizations

The torque measurements after 50 and 100 sterilizations are presented in Table 2 and compared to baseline measurements. The torque after 50 sterilizations in the case of spring-type wrenches was 29.78 ± 14.32 Ncm, and compared to the baseline measurement of 31.95 ± 14.60 Ncm, a significant decrease was confirmed, *p* = 0.006. The mean torque for spring-type wrenches after 100 sterilizations was also lower compared to the baseline (30.07 ± 12.03 Ncm), but not significantly (*p* = 0.117). The respective % changes for spring-type wrenches were −6.78 ± 13.39 and −1.61 ± 17.26. Subtype analysis indicated a significant decrease in torque after 50 sterilizations and after 100 sterilizations in the group of spring-type wrenches set to 45/50 Ncm (*p* = 0.025 and *p* = 0.011, respectively). The evolution (in %) from the baseline was −7.99 ± 7.22 after 50 sterilizations and −13.13 ± 8.39 after 100 sterilizations.

The torque after 50 sterilizations in the case of toggle-type wrenches was 29.78 ± 9.99 Ncm, and compared to the baseline measurement of 28.93 ± 10.05 Ncm, a significant increase was confirmed, *p* = 0.047. The mean torque for toggle-type wrenches after 100 sterilizations was also higher compared to the baseline (29.83 ± 10.71 Ncm), but not significantly (*p* = 0.111). The respective % changes for toggle-type wrenches were 3.32 ± 5.84 and 2.58 ± 7.19.

The mean torque of contra-angle-type wrenches at baseline measurement was 22.99 ± 9.56 Ncm; after 50 sterilizations, it was 24.15 ± 9.24 Ncm; and after 100 sterilizations, it was 23.60 ± 8.95 Ncm. Statistically significant differences between the measurements after the sterilizations and at baseline were not confirmed (*p* = 0.238 and *p* = 0.620 after 50 sterilizations and after 100 sterilizations, respectively).

## 5. Discussion

Torque control plays an important role in implant prosthodontics, and torque controllers play a crucial role. Choosing the right controller is essential, especially in terms of the reliability of its measurements both at its time of purchase and over time. Monitoring the readings of various types of controllers is not easy. The manufacturers and sellers of implant systems rarely provide any service for verifying the correct function of the wrench. Electronic torque verification devices are commercially available, but their usefulness in dental practices is limited; they are either imprecise or very expensive (incomparably more expensive than the cost of a new mechanical torque wrench). Additionally, they often operate in a completely different torque range. The torque generated by controllers in implant prosthodontics is approximately a hundred times lower compared to that of the wrenches used in mechanics (for example, in car workshops). Furthermore, such electronic devices also require control and calibration if they are to be used to verify mechanical devices.

Hence, the idea of constructing devices that use the laws of physics underlying the definition of torque arises. Their construction and function are detailed in Section 3. The required components are inexpensive and readily available (a clamp, a laboratory scale, and weights). The described methodology uses epoxy resin, although self-polymerizing or composite resins could also be used. All the necessary components can be fabricated quickly and easily. For obvious reasons, the design of the devices had to differ slightly depending on the type of torque controller.

The described devices and research methodology are based on the laws of physics and, provided that the measurement precision requirements are met, should not require validation or calibration. The only electronic component in the entire measurement system is a laboratory scale, which is factory-calibrated.

This research is characterized by a certain subjectivity in several aspects. The first relates to the research methodology. In the case of spring-type controllers, this involves a visual assessment of the alignment of the spring arm with the lines on the scale. There are publications [23,24] that describe differences in the interpretation of torque values depending on the viewing angle of the spring-type controller. From a clinical perspective, it is important to mention that the popular spring-type controllers showed relatively poor accuracy in the low-torque range in this study, a range that appears to be very important.

In the case of toggle-type controllers, the wrench clicks across a relatively wide range of torques, making it difficult to determine the specific weight that causes the wrench to “click” [13,14]. Initially, the wrench bends, only to click when the load is increased. Minor movements or vibrations in the ground can lead to premature clicking. During the tests, due care was taken to ensure the stability of the ground on which the test equipment was placed, and the calculation of the test results included the load mass that led not only to deflection but also to actual breakage of the wrench. As mentioned, in order to reduce operator-dependent effects, all procedures were performed by one person throughout the study.

Furthermore, the study omitted the actual mass of the folding key or spring arm. However, measuring the mass of these elements and including them in the test results would have been very cumbersome and seemingly would have had little impact on the results and their interpretation.

The fact that the horizontal arm of the spring wrench deviates from the horizontal plane during measurement, and that its shape takes the form of an arc with a variable chord, was also not taken into account [5]. Nevertheless, all of the above aspects should not have had a significant impact on the quality of the measurements and would have applied equally to all the wrenches in the study group.

Secondly, there is some subjectivity regarding the number of keys examined, but further expansion of the study group seems unjustified, as confirmed by the study’s results and their statistical analysis.

Thirdly, the number of sterilizations (one hundred) is low compared to that in clinical practice, where torque controllers are used for many years and often undergo many more cycles of use and sterilization [2,8,11,18,19,20,25,26,27,28].

It is necessary to mention that torque values on the scales of different types of controllers are divergent and it is impossible to test exactly the same values. Manufacturers’ recommendations vary slightly depending on the type of restoration and the diameter of the screw or implant, but three torque ranges are generally used: low (10–15 Ncm), medium (25–25 Ncm), and high (40–50 Ncm). The research was conducted for the most similar values on the scale, and it should be emphasized that the percentage deviation from the indicated value should be considered the most important parameter.

In summary, it should be emphasized that the results of this study encourage further research on this topic. The use of a relatively small number of application and sterilization cycles indicates no impact on the performance of the torque controllers. However, this does not mean that a higher number of cycles will not affect the precision of their readings in terms of material. For a number of reasons, and considering research that uses a different methodology [29,30], different “aging” technology and different methods for the technical evaluation (e.g., microscopic) could be considered.

Nevertheless, based on the conducted research, the following conclusions can be drawn.

## 6. Conclusions

- The effects of multiple use and sterilization on the accuracy of readings of different types of torque controllers seem limited overall;
- Toggle-type wrenches showed the most stable measurement accuracy;
- The spring-type wrenches most commonly used in practice present the greatest decrease in measurement precision, which should prompt greater caution during their long-term use.

## Figures and Tables

**Table 1:** Torque baseline measurement vs. setting.

Wrench	N	Absolute Torque Value, Ncm Baseline Measurement			Torque Difference to Setting *, %	
		M ± SD	Range	*p*	M ± SD	Range
By subtype						
Spring-type 10/15 Ncm	10	16.20 ± 3.05	11.00–21.94	0.029	16.85 ± 20.94	−2.67–55.20
Spring-type 30/35 Ncm	10	34.86 ± 5.02	30.12–44.58	0.128	9.44 ± 16.91	−13.94–36.70
Spring-type 45/50 Ncm	7	50.30 ± 7.14	41.75–59.64	0.096	8.02 ± 10.87	−7.22–19.28
Toggle-type 20 Ncm	6	20.20 ± 3.87	12.72–22.95	0.905	0.99 ± 19.36	−36.40–14.75
Toggle-type 40 Ncm	6	37.66 ± 4.93	29.03–44.24	0.297	−5.85 ± 12.32	−27.42–10.60
Contra-angle-type 15 Ncm	6	14.77 ± 2.22	12.10–17.40	0.807	−1.56 ± 14.79	−19.33–16.00
Contra-angle-type 35 Ncm	6	31.22 ± 5.80	24.90–41.20	0.171	−10.81 ± 16.57	−28.86–17.71
By type						
Spring-style	27	31.95 ± 14.60	11.00–59.64	0.002	11.82 ± 17.14	−13.94–55.20
Toggle-type	12	28.93 ± 10.05	12.72–44.24	0.420	−2.43 ± 15.88	−36.40–14.75
Contra-angle-type	12	22.99 ± 9.56	12.10–41.20	0.157	−6.18 ± 15.73	−28.86–17.71

M—mean; SD—standard deviation; *p*—significance of difference between baseline measurement and setting of each wrench, based on paired *t* test. * Baseline measurement vs. setting.

**Table 2:** Torque evolution after sterilizations.

Wrench	Absolute Torque Value, Ncm, M ± SD			p1	p2	Torque Difference to Baseline *, %, M ± SD	
	Baseline	After 50 st.	After 100 st.			After 50 st.	After 100 st.
By subtype							
Spring-type 10/15 Ncm	16.20 ± 3.05	14.64 ± 2.39	16.99 ± 2.53	0.096	0.329	−8.09 ± 15.73	6.69 ± 16.36
Spring-type 30/35 Ncm	34.86 ± 5.02	33.16 ± 6.14	33.70 ± 5.54	0.303	0.633	−4.61 ± 15.07	−1.82 ± 19.14
Spring-type 45/50 Ncm	50.30 ± 7.14	46.59 ± 9.60	43.59 ± 7.20	0.025	0.011	−7.99 ± 7.22	−13.15 ± 8.39
Toggle-type 20 Ncm	20.20 ± 3.87	21.13 ± 4.35	20.94 ± 5.04	0.204	0.300	4.46 ± 7.97	2.58 ± 9.02
Toggle-type 40 Ncm	37.66 ± 4.93	38.43 ± 4.61	38.72 ± 6.08	0.167	0.280	2.19 ± 2.88	2.58 ± 5.68
Contra-angle-type 15 Ncm	14.77 ± 2.22	15.55 ± 1.56	15.45 ± 2.19	0.199	0.326	6.23 ± 9.31	5.20 ± 11.79
Contra-angle-type 35 Ncm	31.22 ± 5.80	32.75 ± 2.82	31.75 ± 3.49	0.447	0.834	7.01 ± 16.07	4.20 ± 20.43
By type							
Spring-type	31.95 ± 14.60	29.78 ± 14.32	30.07 ± 12.03	0.006	0.117	−6.78 ± 13.39	−1.61 ± 17.26
Toggle-type	28.93 ± 10.05	29.78 ± 9.99	29.83 ± 10.71	0.047	0.111	3.32 ± 5.84	2.58 ± 7.19
Contra-angle-type	22.99 ± 9.56	24.15 ± 9.24	23.60 ± 8.95	0.238	0.620	6.62 ± 12.53	4.70 ± 15.91

M—mean; SD—standard deviation; 50 st.—after 50 sterilizations; 100 st.—after 100 sterilizations; p1—significance of difference between baseline measurement and measurement after 50 sterilizations, based on paired *t* test; p2—significance of difference between baseline measurement and measurement after 100 sterilizations, based on paired *t* test. * Measurement after sterilizations vs. baseline.

## Data Availability

The raw data supporting the conclusions of this article will be made available by the authors on request.
